# Implementation of a novel process for post-discharge microbiology results review for musculoskeletal infections in a large-volume academic healthcare system

**DOI:** 10.5194/jbji-10-447-2025

**Published:** 2025-11-10

**Authors:** Margaret Pertzborn, Amy L. Van Abel, Trudi Lane, Kristin Cole, Douglas Osmon, Diana J. Schreier, Hilary Teaford, Courtney M. Willis, Anna Woods, Raymund R. Razonable, Abinash Virk, Christina G. Rivera

**Affiliations:** 1 Department of Pharmacy, Mayo Clinic Health System – Northwest Wisconsin Region, Eau Claire, Wisconsin, 54702, United States; 2 Department of Pharmacy, Mayo Clinic, Rochester, Minnesota, 55905, United States; 3 Department of Quantitative Health Sciences, Mayo Clinic, Rochester, Minnesota, 55905, United States; 4 Division of Public Health, Infectious Diseases, Occupational Medicine, Mayo Clinic, Rochester, Minnesota, 55905, United States; 5 Department of Orthopedic Surgery, Mayo Clinic, Rochester, Minnesota, 55905, United States; 6 Department of Pharmacy, Mayo Clinic, Jacksonville, Florida, 32224, United States

## Abstract

Awaiting final microbiology results can delay discharge in musculoskeletal (MSK) infections. We developed a novel process based on electronic medical records reviewing post-discharge results. Among 1662 encounters, 35.6 % had ≥ 1 intervention, often therapy modification. Multidisciplinary review by an orthopaedic infectious diseases team improved antimicrobial optimization through timely action on culture results after discharge.

## 1 Introduction

Transitions of care (TOC) represent a critical juncture where patients are particularly vulnerable to medical errors, (Forster et al., 2003) including those related to microbiology results. Intervention and documentation on delayed microbiology results can occur in > 50 % of relevant post-discharge microbiology results (LaPlante et al., 2023).

Patients with musculoskeletal (MSK) infections may be discharged quickly, prior to the finalization of microbiology results. Some MSK infections involve anaerobes, mycobacterium, and fungi, which can take weeks of incubation. Further, diagnosis of periprosthetic joint infection (PJI) remains challenging and requires understanding of synovial fluid aspiration, diagnostic imaging, peripheral serum inflammatory markers, pathology, and molecular testing in concert with traditional culture (Nodzo et al., 2015). Given the diagnostic intricacies involved with MSK-specific infections, culture review processes in emergency department “culture call backs” or for urine cultures may not directly apply.

This study aimed to investigate the impact and types of post-discharge microbiology results reviewed in MSK infections by a multidisciplinary orthopaedic infectious diseases (ID) team.

## 2 Methods

### 2.1 Study design and setting

Patients from four Mayo Clinic hospital sites from 1 January 2019 through 28 February 2023 were included for retrospective review. Initial implementation of the ID post-discharge microbiology review began 1 January 2019 at the Mayo Clinic, Rochester region, continuing throughout the entirety of the study period. Additional sites were added with the following initiation periods: Mayo Clinic, Florida (1 May 2022); Mayo Clinic Health System, Mankato (1 October 2022); and Mayo Clinic Health System, Eau Claire (1 December 2022).

### 2.2 Participants

Eligible adults ( > 18 years) with an inpatient or observation hospital encounter and an ID consultation in the prior 90 d and with a microbiology result from an MSK infection site (Table S1 in the Supplement) that updated within 24 h prior to or after discharge were included. Minnesota patients without research authorization were excluded (Melton, 1997).

### 2.3 Operational process

An operational report within Epic (Epic Systems Corporation, Verona, WI) was utilized to identify updated microbiology results following patient discharge and was reviewed Monday through Friday; this report flagged abnormal results from the prior 42 d for patients with an inpatient ID consult in the preceding 90 d, with pharmacists documenting review status, interventions as needed, and re-flagging triggered by subsequent result changes. This report is described in detail elsewhere (Van Abel et al., 2024). This study is an MSK-specific subset of previously published work from Van Abel et al. (2024), further chart abstraction was completed for this MSK-specific cohort, and all presented data are new for this subset and abstraction.

### 2.4 Study data process

Retrospective reports based on electronic medical records were utilized to identify patient demographics and interventions. A random subset of 60 MSK infection patients with an intervention, 10 % of overall interventions, was further reviewed and utilized for data abstraction on secondary outcomes, as outlined in Table S2. Data were abstracted utilizing Research Electronic Data Capture (REDCap; Vanderbilt University, Nashville, TN). As previously described in Van Abel et al. (2024), severity ratings for pharmacist interventions were determined by researchers during data collection utilizing a scale modified from the National Coordinating Council for Medication Error Reporting and Prevention Index for categorizing medication errors (Hartwig et al., 1991). Severity ratings were defined as follows: Category 1 – failure to intervene may have resulted in significant patient harm including death or permanent damage; Category 2 – failure to intervene may have resulted in minor or temporary patient harm; Category 3 – intervention resulted in therapy optimization such as de-escalation or decreased costs. A second researcher validated all Category 1 severity ratings.

### 2.5 Statistical analysis

Data were summarized using counts and percentages for categorical data and medians and interquartile ranges (IQRs) or means and standard deviations (SDs) for continuous data. A 95 % binomial exact confidence interval (CI) was calculated for the intervention rate. All analyses were performed using SAS version 9.4 software (SAS Institute, Inc., Cary, NC).

### 2.6 Demographics

A total of 1662 patient encounters with at least one MSK-infection-related microbiology result reviewed post-discharge were identified. Patients were predominantly white and male; additional patient demographics are outlined in Table 1. Osteomyelitis was the most common infection type (51.7 %), and organism identification was the most frequent result type (65 %) in our random sample of 60 encounters. Additional infection characteristics are outlined in Table S3.

**Table 1:** Patient demographics of included encounters (total cohort).

	Total
	( N=1662 )
Age, mean (SD)	61.2 (14.9)
Sex	
Female	632 (38.0 %)
Male	1030 (62.0 %)
Race/ethnicity	
Asian	10 (0.6 %)
Black or African American	38 (2.3 %)
Hispanic or Latino	45 (2.7 %)
White	1525 (91.8 %)
Other	33 (2.0 %)
Unknown	11 (0.7 %)
Region	
Florida region	71 (4.3 %)
MCHS NWWI region	11 (0.7 %)
MCHS SWMN region	17 (1.0 %)
Rochester region	1563 (94.0 %)
LOS (days), median (IQR)	4 (3, 6)
Discharge disposition	
Acute care hospital	11 (0.7 %)
Home or self-care	902 (54.3 %)
Home health care service	515 (31.0 %)
Hospice	4 (0.2 %)
Left against medical advice/discontinued care	10 (0.6 %)
Rehab facility	15 (0.9 %)
Skilled nursing facility	187 (11.3 %)
Transitional care unit	11 (0.7 %)
Other	7 (0.4 %)

Abbreviations: IQR – interquartile range; LOS – length of stay; MCHS – Mayo Clinic Health System; NWWI – Northwest Wisconsin; SD – standard deviation; SWMN – Southwest Minnesota.

## 3 Results

**Figure 1:**
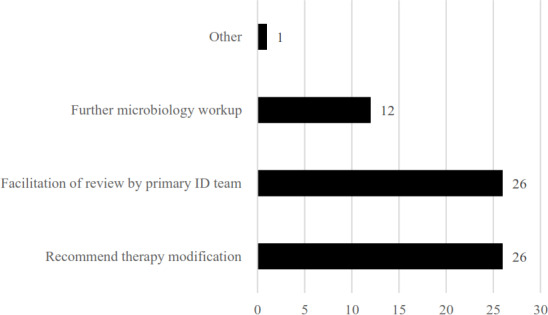
Intervention type(s) ( N=60 ). Note: some reviews had multiple intervention types; therefore the number of intervention types is greater than the number of patients reviewed.

Of the 1662 patient encounters, 592 (35.6 %) had at least one intervention, and a subset of 50 (8.4 %) had ≥ 2 interventions. All other encounters were reviewed independently by an ID pharmacist and deemed not to need further action. A subset of 60 random interventions was further analysed. The most common interventions made were therapy modification (escalation, de-escalation, antimicrobial addition, change in dose or duration) and facilitation of review by primary ID team (consultant, fellow, or advance practice provider (APP)), followed by further microbiology workup (Fig. 1). Depending on the practice site, some interventions (further microbiology workup, dose adjustment) were taken independently by a pharmacist via collaborative practice agreement.

Of the interventions, 10 % (6/60) prevented significant patient harm (Category 1), 25 % (15/60) prevented minor or temporary harm (Category 2), and 16.7 % (10/60) optimized patient care (Category 3). A Category 1 case involved a patient with osteomyelitis discharged on ceftriaxone whose cultures updated post-discharge showed *Pseudomonas aeruginosa*, leading to a switch to cefepime and an extension of therapy to complete 6 weeks of active treatment. In a Category 2 case, a patient with a PJI discharged on daptomycin at 8 mg kg^−1^ had the dose increased to 10 mg kg^−1^ following a culture update showing *Enterococcus faecalis*. A Category 3 example included a patient with a distal femur hardware infection and history of osteosarcoma, initially discharged on doxycycline and cefadroxil, with a recommendation to discontinue doxycycline if *Staphylococcus capitis* was methicillin-susceptible; doxycycline was stopped, and the patient continued cefadroxil. Further details on Category 1 interventions made are outlined in Table S4.

## 4 Discussion

A significant challenge during TOC is managing pending microbiology results, which can delay patient discharge and lead to suboptimal therapy (Forster et al., 2003). Our findings reveal that 35.6 % of MSK-infection-related encounters reviewed post-discharge required intervention, of which 10 % prevented significant patient harm. This intervention rate was higher compared to previous studies focusing on other infectious syndromes, suggesting that MSK infections may present unique challenges that necessitate more frequent involvement, possibly due to the complex nature of diagnosis and management of MSK infections.

Important to these efforts are the multidisciplinary team with multiple professionals involved: ID APPs, pharmacists, and fellows with ID attendings available to these team members, generally for review of more complicated results. Therapy modification was the most common intervention highlighting that a “safety net” report developed, maintained, and operationalized by ID specialists added value.

This study has several limitations that should be considered. Firstly, it was conducted within a single health system, which may limit the generalizability of the findings. The retrospective nature relies on existing records and may not fully capture all relevant details. The analysis of interventions was based on a random sample of only 10 % of total interventions in our population and therefore may not fully represent the broader population. Additionally, this study did not include data on patient clinical outcomes following intervention. Lastly, we could not perform this work as a quasi-experimental study, as discharge culture review in various forms has been a long-standing practice at our institution (Wilson et al., 2011). Further evolution of the existing process could incorporate machine learning to select the results of probable clinical relevance.

## 5 Conclusions

In conclusion, our study demonstrates that a methodical review of post-discharge microbiology results by a multidisciplinary orthopaedic ID team improves the care of patients with MSK infection.

## Supplement

10.5194/jbji-10-447-2025-supplement The supplement related to this article is available online at https://doi.org/10.5194/jbji-10-447-2025-supplement.

## Data Availability

The data that support the finding of this study are available from the corresponding author upon reasonable request.

## References

[bib1.bib1] Forster AJ, Murff HJ, Peterson JF, Gandhi TK, Bates DW (2003). The incidence and severity of adverse events affecting patients after discharge from the hospital. Ann Intern Med.

[bib1.bib2] Hartwig SC, Denger SD, Schneider PJ (1991). Severity-indexed, incident report-based medication error-reporting program. Am J Health-Syst Pharm.

[bib1.bib3] LaPlante R, Claeys KC, Bork JT, Banoub M, Noval M (2023). Evaluating the follow-up of post-discharge positive sterile site cultures and the impact on infection-related complications. Infect Dis Ther.

[bib1.bib4] Melton LJ (1997). The threat to medical-records research. N Engl J Med.

[bib1.bib5] Nodzo SR, Bauer T, Pottinger PS, Garrigues GE, Bedair H, Deirmengian CA, Segreti J, Blount KJ, Omar IM, Parvizi J (2015). Conventional diagnostic challenges in periprosthetic joint infection. J Am Acad Orthop Surg.

[bib1.bib6] Van Abel AL, Virk A, Cole K, Lane T, Osmon D, Pertzborn M, Schreier D, Teaford H, Willis C, Woods A, Rivera CG (2024). Impact of pharmacist-led microbiology result follow-up post-discharge for patients undergoing inpatient infectious diseases consultation. Antimicrob Steward Healthc Epidemiol.

[bib1.bib7] Wilson JW, Marshall WF, Estes LL (2011). Detecting delayed microbiology results after hospital discharge: improving patient safety through an automated medical informatics tool. Mayo Clin Proc.

