# Supplementary material for: Implementation of a novel process for post-discharge microbiology results review for musculoskeletal infections in a large-volume academic healthcare system
Source: J Bone Jt Infect. 2025 Nov 10;10(6):447–50. doi: 10.5194/jbji-10-447-2025 (PMC12628069; doi:10.5194/jbji-10-447-2025)
Supplement: The supplement related to this article is available online at https://doi.org/10.5194/jbji-10-447-2025-supplement. [file jbji-10-447-2025-supplement.pdf]

Supplement of J. Bone Joint Infect., 10, 447–450, 2025
https://doi.org/10.5194/jbji-10-447-2025-supplement
© Author(s) 2025. CC BY 4.0 License.

Supplement of

Implementation of a novel process for post-discharge microbiology results review for musculoskeletal infections in a large-volume academic healthcare system

Margaret Pertzborn et al.

Correspondence to: Margaret Pertzborn (pertzborn.margaret@mayo.edu)

The copyright of individual parts of the supplement might differ from the article licence.

Table S1. Included Microbiology Specimen Types

**Body Region**	**Sample Type**	**Anatomical Site**
Upper Extremity	Bone	Elbow, Finger, Hand, Ulna, Shoulder, Wrist
	Tissue	Elbow, Humerus, Ulna, Wrist
	Fluid	Elbow, Finger, Hand, Shoulder, Synovial fluid (Elbow, Shoulder)
	Swab	Finger, Hand, Wrist
	Prosthetic Material / Implant	Elbow, Shoulder
	Drainage	Finger, Hand, Wrist
Lower Extremity	Bone	Fibula, Tibia
	Tissue	Ankle, Hip, Knee
	Fluid	Hip, Synovial fluid (Ankle, Hip, Knee)
	Swab	Ankle, Hip
	Prosthetic Material / Implant	Hip, Knee
Spine	Bone	Sacrum
	Tissue	Intervertebral disc, Spine
	Fluid	Back, Spine
	Swab	Spine
	Prosthetic Material / Implant	Spine
Pelvis	Bone	Ilium, Ischium, Pelvis
	Tissue	Ischium
Other	Prosthetic Joint / Orthopaedic Implant	

Table S2. Outcome Assessment

	POPULATION ASSESSED	
	Entire Cohort (n=1662)	Sample of RPh Interventions (n=60)
**Primary Outcome**		
% of abnormal patient encounters requiring RPh intervention	x	
**Secondary Outcomes**		
Time from result update to RPh review	x	
RPh intervention types		x
RPh intervention severity		x
Microbiology result type		x
Type of organism identified		x
**Patient Characteristics**		
Age	x	
Gender	x	
Race	x	
Length of hospital stay	x	
Type of infection	x	

Abbreviations: RPh-pharmacist

Table S3: Infection Characteristics (Focused Review Subset)

	Total (N=60)
**Infection Type**	
Osteomyelitis	31 (51.7%)

	Total (N=60)
Periprosthetic joint infection	30 (50.0%)
Skin and soft tissue infection	2 (3.3%)
**Microbiology Result Type**	
Organism identification	39 (65.0%)
Susceptibility result – agar dilution	12 (20.0%)
Susceptibility result - automated	4 (6.7%)
Polymerase Chain Reaction result	7 (11.7%)
Gram stain	3 (5.0%)
Other	1 (1.7%)
**Type of Organism Associated with Abnormal Result**	
Aerobic gram-positive bacteria	30 (50.0%)
Anaerobic bacteria	19 (31.7%)
Aerobic gram-negative bacteria	8 (13.3%)
Fungal – yeast	8 (13.3%)
Fungal – mold	2 (3.3%)
**Specimen Source**	
Bone	8 (13.3%)
Tissue	43 (71.7%)
Tissue swab	3 (5%)

	Total (N=60)
Blood	1 (1.7%)
Body fluid	3 (5%)
Joint aspirate	3 (5%)
Prosthetic material	1 (1.7%)
Other	1 (1.7%)

Note: Patients may fall into multiple categories for infection type and other fields; therefore, totals may equal or exceed 100%.

Abbreviations: ID - Infectious Diseases

Table S4. Interventions with Category 1 Severity Rating Details

Intervention Number	Infection	Intervention Type	Intervention Description
1	Osteomyelitis	Recommend therapy modification	Patient with left greater trochanteric wound s/p left gluteus myocutaneous flap was discharged on oral sulfamethoxazole-trimethoprim with planned duration of 8 weeks of therapy. Intraoperative tissue and bone cultures grew *Fusobacterium* spp. and *Peptoniphilus* spp. post discharge. The pharmacist recommended oral amoxicillin-clavulanate for anaerobic coverage which was initiated.
2	Periprosthetic Joint Infection	Recommend therapy modification and further microbiology work up	Patient with failed revision reverse shoulder arthroplasty s/p resection arthroplasty and spacer placement was discharged on 14 days of oral cefadroxil with plan to extend the treatment duration if cultures became positive. The pharmacist reviewed intraoperative tissue cultures that resulted post discharge as *Cutibacterium acnes*, requested antimicrobial susceptibilities, and facilitated extension of oral cefadroxil to 6 weeks.
3	Periprosthetic Joint Infection	Recommend therapy modification	Patient on chronic suppression with oral amoxicillin-clavulanate for right total hip arthroplasty periprosthetic joint infection was found to have a periprosthetic joint infection following left total hip

			arthroplasty. Patient was discharged on oral doxycycline and continued on amoxicillin-clavulanate. Intraoperative tissue cultures grew pan-susceptible *Pseudomonas aeruginosa*, and the pharmacist recommended initiation of oral ciprofloxacin. Broad range PCR also later resulted as *P. aeruginosa* and *Serratia spp.,* which was reviewed by pharmacist and ciprofloxacin was continued.
4	Periprosthetic Joint Infection	Further microbiology workup	Patient undergoing revision of left total hip arthroplasty had an unexpected positive intraoperative tissue culture while receiving oral doxycycline 100 mg twice daily. Post-discharge, the isolate was identified as *Siminovitchia fordii*, susceptible to levofloxacin, sulfamethoxazole-trimethoprim, and vancomycin, but resistant to clindamycin and penicillin. Tetracycline susceptibility was not reported. The pharmacist intervened to request tetracycline susceptibility testing, which confirmed susceptibility.
5	Osteomyelitis	Recommend therapy modification	Patient with chronic right foot infection s/p irrigation and debridement, with history of prolonged QTc, had an intraoperative tissue culture positive for *Candida albicans*. Patient was discharged on IV vancomycin. A second operative culture post-discharge also grew *C. albicans*, prompting pharmacist intervention and initiation of oral isavuconazonium sulfate with continuation of IV vancomycin.
6	Osteomyelitis	Recommend therapy modification	Patient with chronic osteomyelitis of the right femur s/p hardware removal, irrigation and debridement, and placement of antibiotic-coated nail and beads was discharged on 6 weeks of IV ceftriaxone after initial negative cultures. Following discharge, the pharmacist identified two intraoperative tissue cultures positive for *P. aeruginosa* and intervened to change therapy to IV cefepime for 6 weeks. The pharmacist also verified susceptibility results once available, confirming *P. aeruginosa* was cefepime-susceptible.

Abbreviations: IV -intravenous, PCR - polymerase chain reaction, s/p - status post, QTc - Corrected QT interval
